# The Integrative Analysis Identifies Three Cancer Subtypes and Stemness Features in Cutaneous Melanoma

**DOI:** 10.3389/fmolb.2020.598725

**Published:** 2021-02-16

**Authors:** Xiaoran Wang, Qi Wan, Lin Jin, Chengxiu Liu, Chang Liu, Yaqi Cheng, Zhichong Wang

**Affiliations:** ^1^ State Key Laboratory of Ophthalmology, Zhongshan Ophthalmic Center, Sun Yat-Sen University, Guangzhou, China; ^2^ The First Affiliated Hospital of Shandong First Medical University, Shandong, China; ^3^ Department of Ophthalmology, Affiliated Hospital of Qingdao University Medical College, Qingdao, China

**Keywords:** cutaneous melanoma, classification, stemness feature, prognosis, canccer stem cell

## Abstract

**Background:** With the growing uncovering of drug resistance in melanoma treatment, personalized cancer therapy and cancer stem cells are potential therapeutic targets for this aggressive skin cancer.

**Methods:** Multi-omics data of cutaneous melanoma were obtained from The Cancer Genome Atlas (TCGA) database. Then, these melanoma patients were classified into different subgroups by performing "CancerSubtypes" method. The differences of stemness indices (mRNAsi and mDNAsi) and tumor microenvironment indices (immune score, stromal score, and tumor purity) among subtypes were investigated. Moreover, the Least Absolute Shrinkage and Selection Operator (LASSO) and Support Vector Machine-Recursive Feature Elimination (SVM-RFE) algorithms were performed to identify a cancer cell stemness feature, and the likelihood of immuno/chemotherapeutic response was further explored.

**Results:** Totally, 3 specific subtypes of melanoma with different survival outcomes were identified from TCGA. We found subtype 2 of melanoma with the higher immune score and stromal score and lower mRNAsi and tumor purity score, which has the best survival time than the other subtypes. By performing Kaplan–Meier survival analysis, we found that mRNAsi was significantly associated with the overall survival time of melanomas in subtype 2. Correlation analysis indicated surprising associations between stemness indices and subsets of tumor-infiltrating immune cells. Besides, we developed and validated a prognostic stemness-related genes feature that can divide melanoma patients into high- and low-risk subgroups by applying risk score system. The high-risk group has a significantly shorter survival time than the low-risk subgroup, which is more sensitive to CTLA-4 immune therapy. Finally, 16 compounds were screened out in the Connectivity Map database which may be potential therapeutic drugs for melanomas.

**Conclusion:** Thus, our finding provides a new framework for classification and finds some potential targets for the treatment of melanoma.

## Introduction

Melanoma is a quite lethal tumor once it has spread (metastasized). Melanoma arises from the precursor lesion with an accumulation of unrestrained mutations; orthotopic melanoma can be cured by resection in combination with continuously proven adjuvant therapy (Bray et al., 2018; Siegel et al., 2019).

Progression of melanoma can be characterized by the genetically distinct subpopulations which are related to a high occurrence of chemotherapy resistance. Given that about 90% of metastatic tumors develop resistance, a high incidence of melanoma in the reduced overall survival rate is due to the resistance to chemotherapies (Chow et al., 2011). At present, there are some validated adjuvant treatments for melanoma. Still, considering the side effects and different drug treatment responses of melanoma patients, the best choice and implementation of comprehensive melanoma therapy are unresolved. It is critical to find a more targeted selection for advanced melanoma patients.

Among tumor cells, the strong chemoresistance of tumor stem cells is closely related to high mortality after metastasis. Cancer stem cells are defined as the precursors by tumorigenesis, self-renewal, and pluripotency, namely, a subset of tumor-initiating cells (Abbaszadegan et al., 2017). To date, melanoma stem cells have been identified as a subpopulation of melanoma cells which can express cellular markers, like CD271, CD133, ABCB5, MDR1, etc. (Civenni et al., 2011; Keshet et al., 2008; Sharma at al., 2010). According to recent studies, melanoma stem cells can participate in related signal transduction pathways and play vital roles in escaping from immune surveillance and resistance to radiation therapy or chemotherapy (El-Khattouti et al., 2014; Mohme et al., 2017; Pak et al., 2004). Studies have found that molecules related to the expression of stem markers in tumors can enhance the resistance of tumors to chemotherapy, which is the basis for cancer stem cells to resist the toxic effects of chemotherapy drugs. The expression level of some stem cell-related markers is positively correlated with chemotherapy tolerance. The reason why cancer stem cells can escape from the cytotoxic effect of chemotherapeutic drugs includes their drug excretion mechanism, anti-apoptosis mechanism, and DNA damage repair mechanism (Meng et al., 2014; Schoning et al., 2017). Cancer stem cells also could express stronger stem cell-related potentials when they resist chemotherapy by activating specific pathways (Takeda et al., 2016). Therefore, the study of the characteristics of drug resistance mechanism of cancer stem cells has excellent application prospects and significance, and it is meaningful for complementary drug treatment programs to melanoma patients.

Current therapeutic strategies targeting tumor stem cells mainly include targeting specific surface markers or intracellular signal transduction pathways, inducing tumor stem cell differentiation, and changing the tumor stem cell microenvironment (Pei et al., 2020; Qin et al., 2020; Zhang et al., 2020). However, some studies have shown that tumor cells can be dedifferentiated into tumor stem cells to supplement depleted tumor stem cells under the influence of their surrounding environment. The ability of this new tumor stem cell to tolerate chemotherapy is still unknown. The heterogeneity of tumors and the complexity of the surrounding microenvironment make tumor treatment extremely complicated, so understanding the tumor heterogeneity and its external environment is vital (Lian et al., 2019). In particular, changes in the immune environment related to tumors will help us further to understand the melanoma therapeutic strategy.

## Materials and Methods

### Data Collection and Cancer Subtype Identification

The transcriptome profile of RNA sequencing data and matched DNA methylation data of cutaneous melanoma as well as clinical information were obtained from the TCGA database. After data processing like distribution check, imputation, and normalization, three data types including gene expression, miRNA expression, and DNA methylation merged into a final dataset for integrative analysis. Next, these melanoma patients were divided into different subgroups by performing three clustering methods in R package ("CancerSubtypes").

### Stemness Index Calculation

Stemness Index Workflow (https://bioinformaticsfmrp.github.io/PanCanStem_Web/) provides the steps and processes to regenerate our stemness indices (mRNAsi and mDNAsi), which train a stemness signature using normal stem cells and apply the one-class algorithm to define a stemness index for each tumor sample. The mRNA stemness index based on a gene set contains 11,774 genes, and the DNA stemness index calculated by a DNA methylation set contains 151 differentially methylated CpG sites. We first scored melanoma patients by applying Stemness Index Workflow and then scaled the stemness indices range from 0 to 1.

### Tumor Microenvironment Estimation

The immune score, stromal score, and tumor purity were calculated from gene expression data by applying the ESTIMATE algorithm in R package (“ESTIMATE”). By running the ESTIMATE algorithm, immune score, stromal score, and tumor purity of each melanoma patient can be estimated. Then, we also scaled the value of immune score, stromal score, and tumor purity range from 0 to 1.

### Evaluation of the Relationship Between Subtype and Clinical Variables

To clarify the clinicopathologic characteristics of the cancer subtypes, the subgroup analysis of clinical variables including mRNAsi, mDNAsi, immune score, stromal score, tumor purity, age, sex, and metastatic status was performed. Next, Kaplan–Meier plots were used to explore the prognostic value of stemness index (mRNAsi and mDNAsi) and found that only mRNAsi had a significant association with overall survival time in all melanoma patients. Hence, mRNAsi was screened out for further analysis. Afterwards, each subtype of melanoma was divided into low and high mRNAsi groups by median cutoff of value, and Kaplan–Meier plots were drawn. The differences between low and high mRNAsi groups in subtypes were compared by log-rank tests. Eventually, Kaplan–Meier survival analysis showed that the mRNAsi was only significantly associated with overall survival in subtype 2. In addition, the cutaneous melanoma patients in subtype 2 were randomly divided into a 70% training dataset and a 30% validation dataset. In training datasets, samples were divided into high and low mRNAsi groups. “Limma” package in R software was applied to identify the differentially expressed genes (DEGs). The |log 2 fold change (FC)| ≥0.5 and *p* values <0.05 were considered as the cutoff criterion for DEGs. Then, univariate Cox regression analysis was used to screen the prognostic DEGs (*p* values <0.05). Next, for subsequently selecting the important mRNAsi-related features, the Least Absolute Shrinkage and Selection Operator (LASSO) and Support Vector Machine-Recursive Feature Elimination (SVM-RFE) algorithms were applied to reduce the prognostic DEGs.

### Identification and Validation of Stemness Features

LASSO and SVM-RFE algorithms jointly determine the qualified seed of DEGs for the risk formula, and the risk score is generated as follows: risk score = $\sum_{i=1}^{\text{N}}\left(coef_{i}\times expr_{i}\right)$, in which N means the number of feature genes, *expr*_*i*_ means the expression level of genes, and *coef*_*i*_ means regression coefficient calculated by multivariate Cox regression analysis.

The risk score of each sample in training dataset was estimated, and the patients were accordingly classified into high- and low-risk group by the median cutoff. Univariate and multivariate Cox logistic analyses for OS were performed on the patient clinical characteristics (age, gender, stage, and metastasis) and the risk score of stemness features.

To compare the differences between high- and low-risk groups, we drew Kaplan–Meier survival curves and calculated the significance by log-rank tests. The area under the curve (AUC) of receiver operating characteristic curves (ROC) was used to evaluate the 5-year overall survival predictive accuracy of the model. Besides, to test the robustness of our results, stemness features were further verified in a validation dataset (GSE65904) which was downloaded from the GEO database.

### Evaluation of the Association Between Stemness Indices and Immune Microenvironment

To explore the relationship between stemness indices and immune microenvironment in different melanoma subtype, single sample gene set enrichment analysis (ssGSEA) method in R package (“GSVA”) was applied to specifically discriminate 24 human immune cells, including innate and adaptive immune cells. The innate immune cells contain natural killer (NK) cells, CD56bright NK cells, CD56dim NK cells, dendritic cells (DCs), activated DCs (aDCs), immature DCs (iDCs), plasmacytoid DCs (pDCs), neutrophils, macrophages, eosinophils, and mast cells, and the adaptive immune cells, including T cells, B cells, and cytotoxic cells. Moreover, the T cells consist of T effector memory (Tem), T central memory cells (Tcm), CD8 T cells, Tgd cells, regulatory T cells (Treg), T helper cells and T follicular helper cells (TFH), Th1, Th2, and Th17. Next, the correlation analysis between stemness indices (mRNAsi/mDNAsi) and 24 immune cells expression was performed.

### Immuno/Chemotherapeutic Response Prediction

To explore the potential immuno/chemotherapeutic drugs, we predicted the candidate compounds response for each sample based on the Connectivity Map website (https://portals.broadinstitute.org/cmap/). The significant compounds were selected (*p *< 0.05). Additionally, immune checkpoint inhibitors have been approved as routine drugs for melanoma. Thus, we also predicted the potential response to immunotherapy by using the TIDE website tool (http://tide.dfci.harvard.edu/).

### Statistical Analysis

All statistical analyses were conducted using the R package (v.3.5.2) and corresponding packages. Survival analysis was applied by using “survival” and “survivalROC” package. LASSO algorithm was conducted by “glmnet” package. SVM algorithm was calculated with the “e1017” package. The correlation coefficient was calculated by Spearman test. For comparisons of two groups and more than two groups, Kruskal–Wallis test and one-way analysis of variance were used as non-parametric and parametric methods, respectively. The association between subgroup and clinicopathological characteristics was analyzed with the chi-square test.

## Results

### Data Collection and Cancer Subtype Identification

**FIGURE 1:**
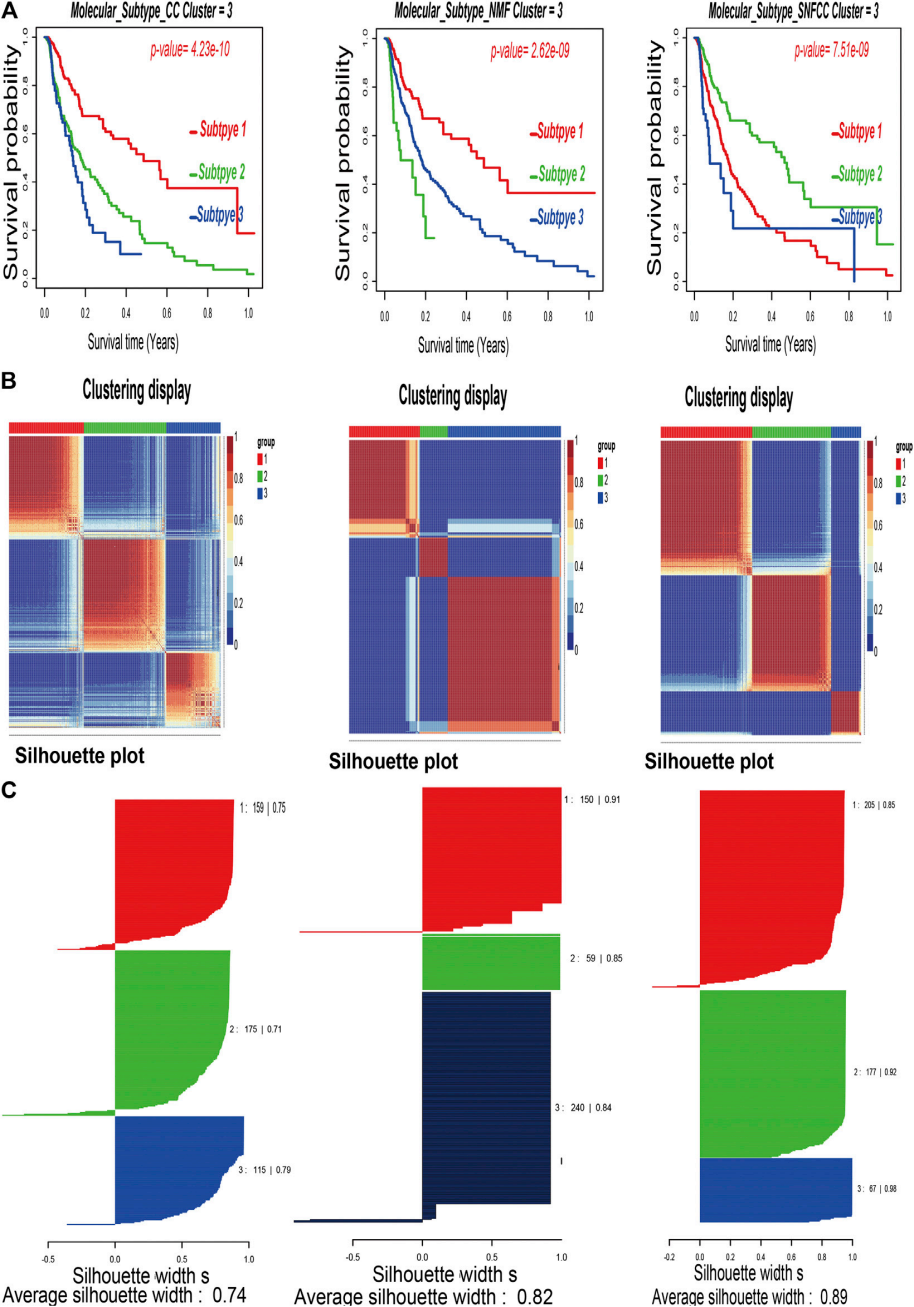
Classification of melanoma patients by three clustering methods including consensus clustering (CC), consensus non-negative matrix factorization (CNMF), and similarity network fusion with CC (SNFCC). **(A)**: Kaplan–Meier survival analysis of three subtypes with log-rank test *p* value; **(B)**: clustering heatmap of three subtype samples; **(C)**: average silhouette width representing the coherence of clusters.

After combining multi-omics data into integrative analysis, 449 melanoma patient samples were obtained from the TCGA database. Then, according to prior studies, these patients were divided into three subtypes by three clustering methods including consensus clustering (CC), consensus non-negative matrix factorization (CNMF), and similarity network fusion with CC (SNFCC) (Lu et al., 2018). Although all the clustering methods can classify melanoma patients into 3 subtypes with different survival outcomes (CC: *p* value = 4.23e-10; NMF: *p* value = 2.62e-09; SNFCC: *p* value = 7.51e-09) (Figure 1A) and clear boundaries between different color areas (Figure 1B), combined with the value of average silhouette width (ASW) which works as a measure of cluster coherence to assess whether samples are more similar within subtypes. SNFCC showed more advantages than other methods and were selected for subsequent analysis (CC: ASW = 0.74; CNMF: ASW = 0.82; SNFCC: ASW = 0.89) (Figure 1C). In SNFCC, subtype 1 contains 205 samples, subtype 2 consists of 177 samples, and subtype 3 includes 67 samples. Among three subtypes, subtype 2 has the longest survival time compared to others.

### Clinicopathologic Characteristics of the Cancer Subtypes

**FIGURE 2:**
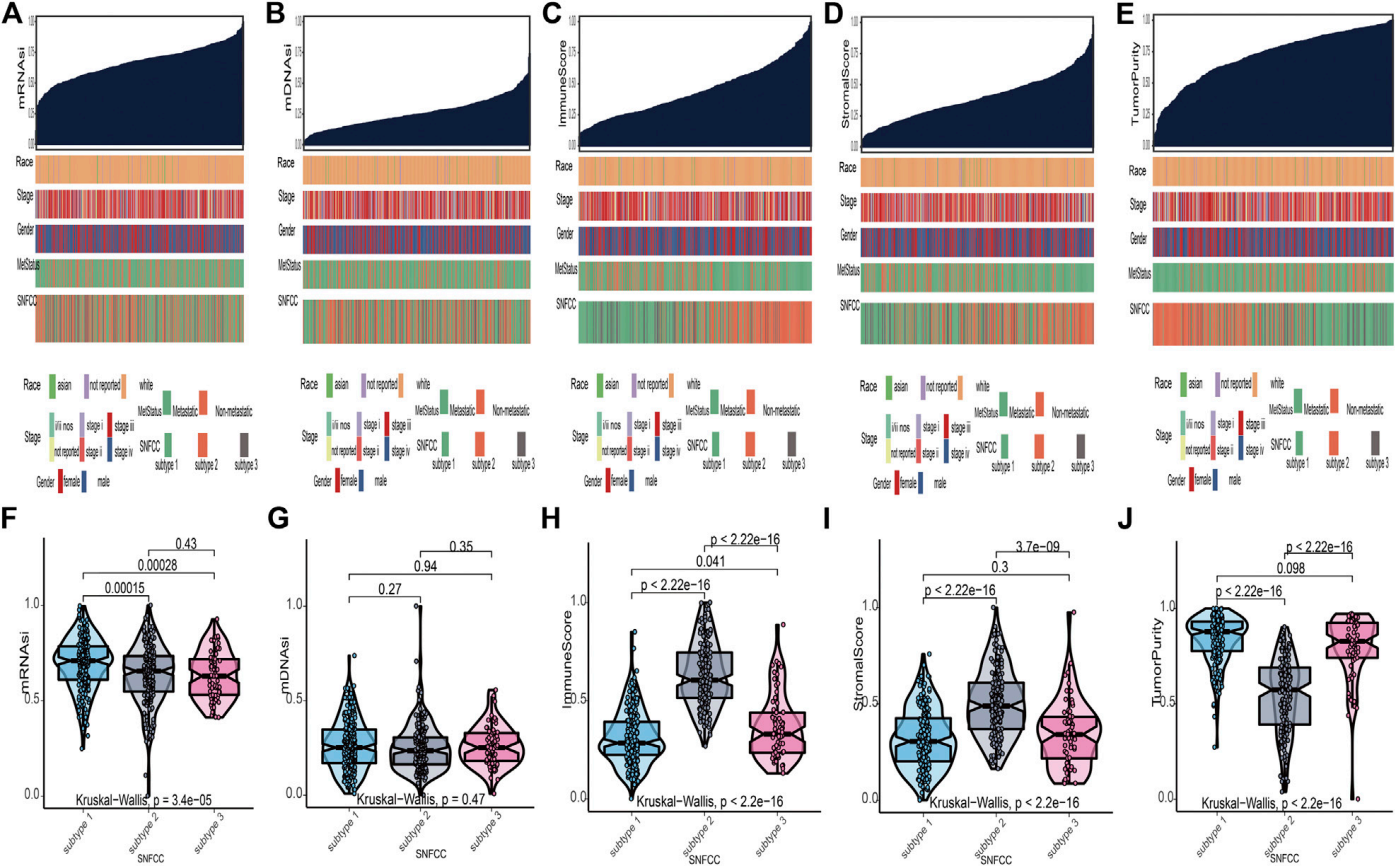
Clinical variables associated with the stemness indices (mRNAsi and mDNAsi) and tumor microenvironment indices (immune score, stromal score, and tumor purity) in melanoma. **(A)**: the association between clinical variables (race, stage, gender, metastatic status, and subtype) and mRNAsi; **(B)**: the association between clinical variables and mDNAsi; **(C)**: the association between clinical variables and immune score; **(D)**: the association between clinical variables and stromal score; **(E)**: the association between clinical variables and tumor purity. Columns represent samples sorted by score of indices from low to high **(top row)**. Rows represent clinical variables. **(F)**: boxplots of mRNAsi in individual samples stratified by subtype; **(G)**: boxplots of mDNAsi in individual samples stratified by subtype; **(H)**: boxplots of immune score in individual samples stratified by subtype; **(I)**: boxplots of stromal score in individual samples stratified by subtype; **(J)**: boxplots of tumor purity in individual samples stratified by subtype.

According to the methods, we acquired stemness indices (mRNAsi and mDNAsi) and tumor microenvironment indices (immune score, stromal score, and tumor purity) of 449 melanoma patients. After excluding adjacent, duplicated, and incomplete samples, data of 427 patients were included for further subgroup analysis. Firstly, the melanoma patients were ordered by their values of stemness and tumor microenvironment indices (from low to high) to explore whether any clinical feature was associated with these calculated indices (Figures 2A–E). Remarkably, the patients in subtype 1 had higher value of mRNAsi (median value = 0.71) than subtype 2 (median value = 0.66) and subtype 3 (median value = 0.63) patients (Table 1). Boxplots of mRNAsi suggested that there is a significant difference among subtypes (Figure 2F). Similarly, subgroup analysis of tumor purity showed that patients in subtype 1 (median value = 0.88) had higher values than subtype 2 (median value = 0.57) and subtype 3 (median value = 0.83) (Figure 2J and Table 1). As for immune and stromal score, results manifested that subtype 2 samples had higher values (immune median value = 0.61; stromal median value = 0.49) than subtype 1(immune median value = 0.28; stromal median value = 0.30) and subtype 3 (immune median value = 0.33; stromal median value = 0.34) (Figures 2H,I and Table 1). However, there is no statistical difference among the three subtypes in mDNAsi index (Figure 2G). The median values of three subtypes were 0.25, 0.24, and 0.25, respectively (Table 1). Next, the subgroup analysis of other clinical variables like overall survival time, age, gender, race, metastatic status, and stages was also applied. The results showed that survival time, age, metastatic status, and stages were statistically different among melanoma subtypes (Table 1).

**TABLE 1:** Clinicopathological variables of subtypes in melanoma. IQR means interquartile range.

		Subtype 1	Subtype 2	Subtype 3	*p*	Test
n		195	167	65		
Survival time (median [IQR])		2.84 [1.30, 5.88]	4.44 [2.27, 9.48]	1.28 [1.01, 2.18]	0.000	Kruskal–Wallis test
Age (median [IQR])		60.00 [49.00, 70.00]	55.00 [45.00, 68.50]	63.00 [56.00, 76.00]	0.002	Kruskal–Wallis test
Gender (%)	Female	64 (32.8)	69 (41.3)	27 (41.5)	0.191	Chi-square test
	Male	131 (67.2)	98 (58.7)	38 (58.5)		
Race (%)	Asian	4 (2.1)	4 (2.4)	4 (6.2)	0.459	Chi-square test
	Not reported	5 (2.6)	3 (1.8)	2 (3.1)		
	White	186 (95.4)	160 (95.8)	59 (90.8)		
MetStatus (%)	Metastatic	159 (81.5)	155 (92.8)	15 (23.1)	0.000	Chi-square test
	Non-metastatic	36 (18.5)	12 (7.2)	50 (76.9)		
Stage (%)	I/II nos	4 (2.1)	5 (3.0)	1 (1.5)	0.000	Chi-square test
	Not reported	13 (6.7)	18 (10.8)	3 (4.6)		
	Stage I	30 (15.4)	40 (24.0)	3 (4.6)		
	Stage II	59 (30.3)	30 (18.0)	40 (61.5)		
	Stage III	77 (39.5)	67 (40.1)	15 (23.1)		
	Stage IV	12 (6.2)	7 (4.2)	3 (4.6)		
mRNAsi (median [IQR])		0.71 [0.61, 0.79]	0.66 [0.55, 0.73]	0.63 [0.53, 0.72]	0.000	Kruskal–Wallis test
mDNAsi (median [IQR])		0.25 [0.17, 0.35]	0.24 [0.16, 0.31]	0.25 [0.18, 0.33]	0.533	Kruskal–Wallis test
Stromal score (median [IQR])		0.30 [0.20, 0.43]	0.49 [0.37, 0.61]	0.34 [0.22, 0.43]	0.000	Kruskal–Wallis test
Immune score (median [IQR])		0.28 [0.22, 0.39]	0.61 [0.51, 0.75]	0.33 [0.24, 0.44]	0.000	Kruskal–Wallis test
Tumor purity (median [IQR])		0.88 [0.78, 0.93]	0.57 [0.39, 0.69]	0.83 [0.74, 0.93]	0.000	Kruskal–Wallis test

### Relationship Between Stemness Indices and Tumor Microenvironment

**FIGURE 3:**
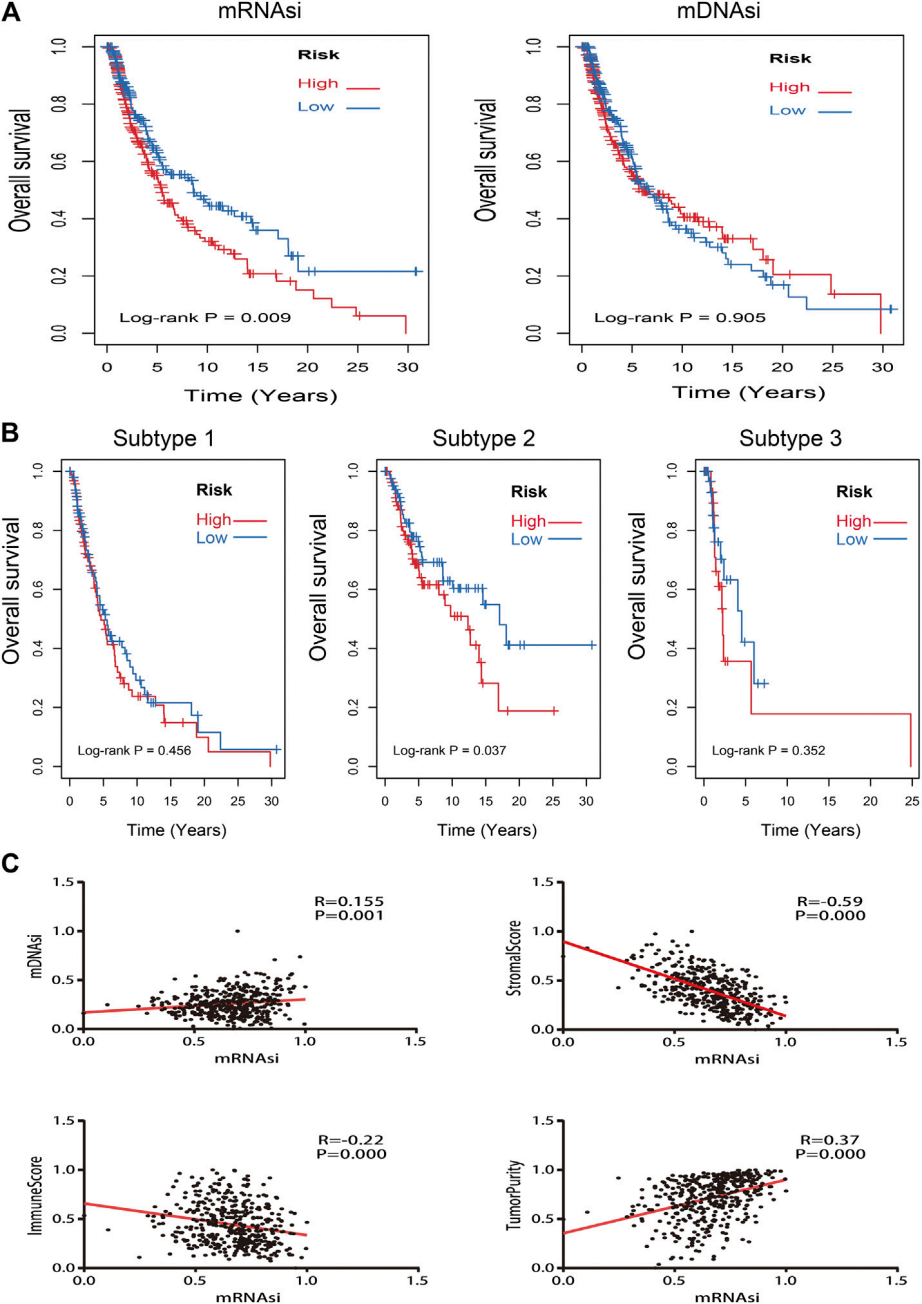
Kaplan–Meier survival analysis and correlation analysis of stemness indices. **(A)**: Kaplan–Meier analysis of mRNAsi and mDNAsi in all melanoma samples; **(B)**: Kaplan–Meier analysis of each subtype of melanoma patients with high or low mRNAsi; **(C)**: the correlation analysis between mRNAsi and other indices (mDNAsi, immune score, stromal score, and tumor purity).

Kaplan–Meier curves of mRNAsi and mDNAsi manifested that only mRNAsi was significantly associated with overall survival time in all melanoma patients, and low mRNAsi group had a longer survival time than high mRNAsi group (log-rank *p* = 0.009) (Figure 3A). Therefore, mRNAsi was selected out for the next analysis. Subgroup analysis of mRNAsi showed that subtype 2 was significantly correlated to overall survival time (log-rank *p* = 0.037), whereas Kaplan–Meier curves of subtype 1 and subtype 3 showed that there was no statistical difference (Figure 3B). In addition, correlation analysis revealed that mRNAsi was positively correlated with mDNAsi (r = 0.155, *p* = 0.001) and tumor purity (r = 0.370, *p* = 0.000), while immune and stromal score were negatively associated with mRNAsi (r = −0.220, *p* = 0.000; r = −0.590, *p* = 0.000) (Figure 3C).

### Identification and Validation of Stemness Features

**FIGURE 4:**
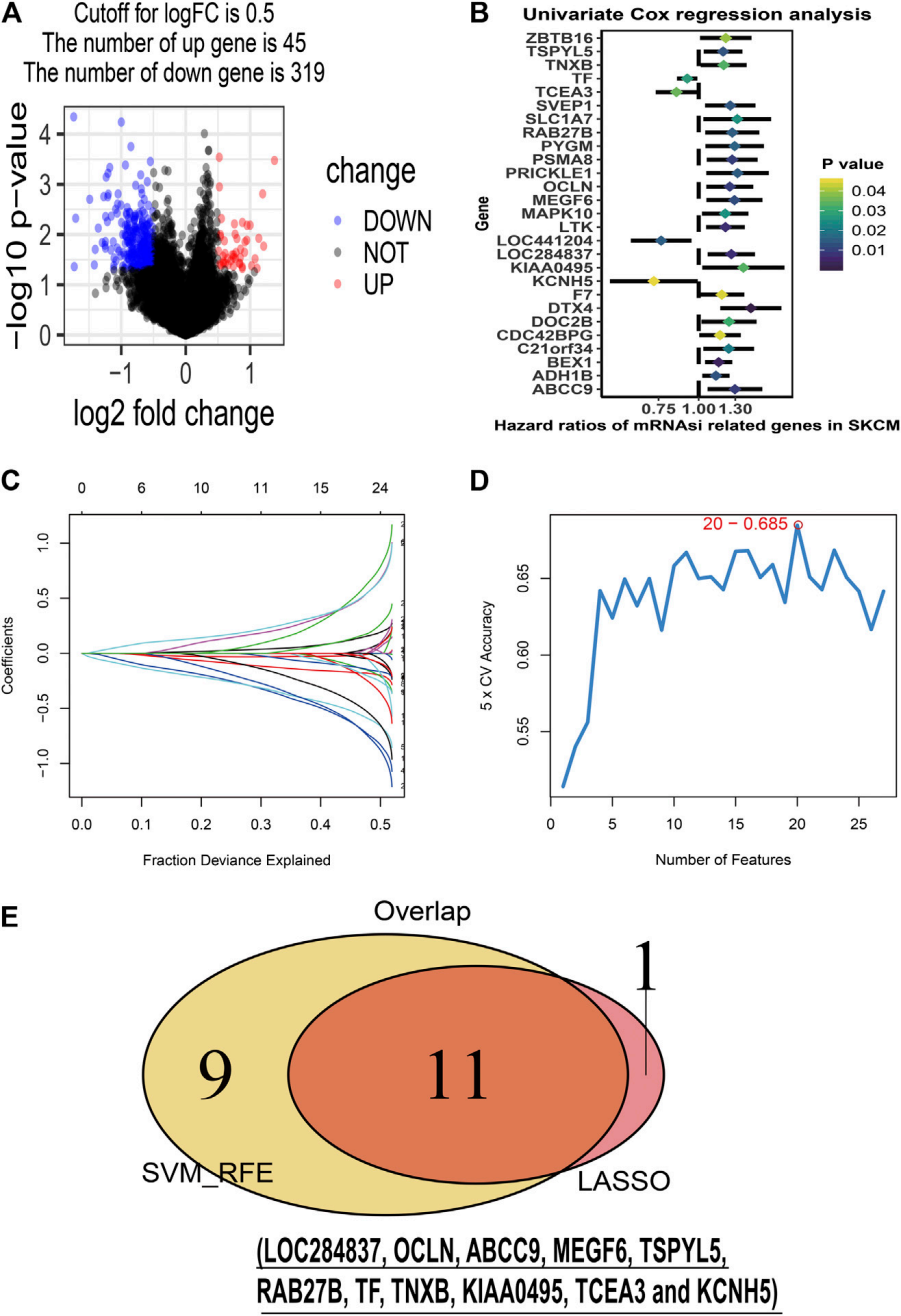
Stemness-related genes feature selection. **(A)**: volcano plot of the differentially expressed stemness-related genes in training dataset; **(B)**: forest plots of the prognostic differentially expressed stemness-related genes; **(C)**: the Least Absolute Shrinkage and Selection Operator (LASSO) algorithm coefficient profiles of the 12 genes that met the prognostic criteria initially; **(D)**: Support Vector Machine-Recursive Feature Elimination (SVM-RFE) algorithms. The point highlighted indicates the lowest error rate, and the corresponding genes at this point are the best signature selected by SVM-RFE. **(E)**: the Venn plot of overlap genes selected by LASSO and SVM-RFE algorithms.

**FIGURE 5:**
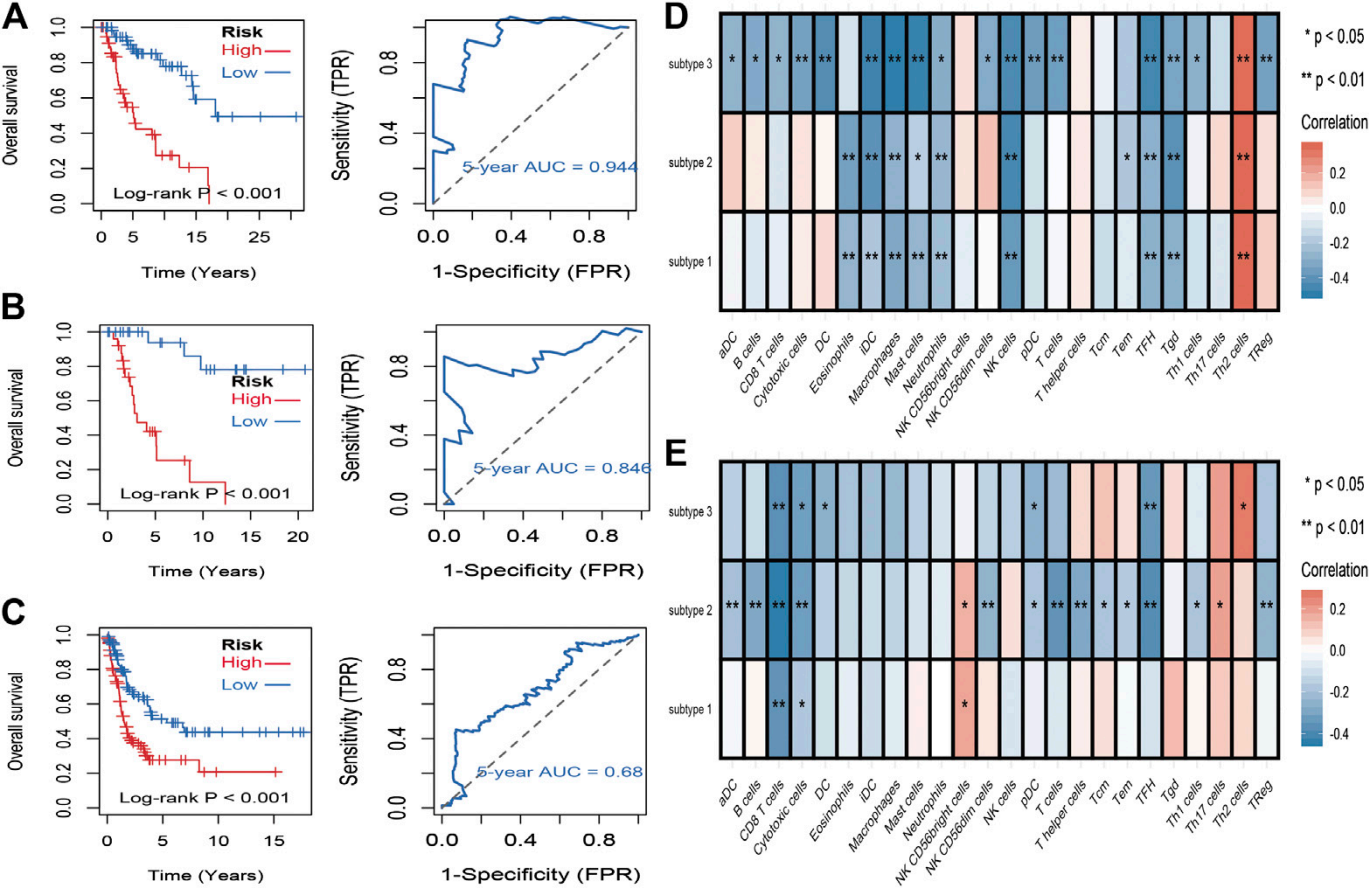
Identification and validation of stemness-related genes feature for survival prediction. **(A)**: Kaplan–Meier analysis of 11 mRNAsi-related genes’ signature and 5 years of the receiver operating characteristic (ROC) curve in training dataset. **(B)**: Kaplan–Meier analysis of 11 mRNAsi-related genes’ signature and 5 years of the receiver operating characteristic (ROC) curve in validation dataset. **(C)**: Kaplan–Meier analysis of 11 mRNAsi-related genes’ signature and 5 years of the receiver operating characteristic (ROC) curve in GSE65904 dataset. **(D)**: correlations between the mRNAsi and the subsets of tumor-infiltrating immune cells estimated by “ssGSEA” method. **(**F**)**: correlations between the mDNAsi and the subsets of tumor-infiltrating immune cells estimated by “ssGSEA” method.

To identify stemness features, subtype 2 samples were randomly divided into a training dataset (n = 117) and a validation dataset (n = 50). The clinical characteristics of training and validation datasets are listed in Table 2, and statistical results indicated that they were balanced between two datasets. Firstly, based on the selection criteria, 364 DEGs were screened out in training dataset, in which 319 genes were significantly downregulated and 45 genes were significantly upregulated (Figure 4A). Next, the univariate analysis of 364 DEGs was conducted, and the results showed that 27 prognostic DEGs were significantly associated with overall survival time in the training dataset (Figure 4B). Finally, 11 mRNAsi-related genes were selected by performing LASSO and SVM-RFE algorithm, and these genes were further used to construct a risk score system (Figures 4C–E). By applying this risk model, a risk score for each sample in the training dataset will be generated. Then, melanoma patients were divided into a high-risk group (n = 58) and a low-risk group (n = 59) by using the median cutoff value of the risk scores. Kaplan–Meier curves showed that patients in the high-risk group have a shorter survival time than low-risk group with a log-rank test of *p* < 0.001. To estimate the prediction power of 11 mRNAsi-related genes’ signature, the ROC curve was drawn, and five years of AUC was 0.944 (Figure 5A). Besides, in order to confirm the robustness of the result, a verification test was conducted in the validation dataset and GSE65904 dataset. The validation and GSE65904 datasets were classified into high-risk and low-risk groups according to the training dataset. Kaplan–Meier curves showed that there is a significant difference between high-risk and low-risk groups in both validation dataset (log-rank *p* < 0.001) and GSE65904 dataset (log-rank *p* < 0.001) (Figure 5B and Figure 5C). The five years of AUC were 0.846 and 0.680, respectively. What is more, to explore the prognostic value of risk score and other clinical features (age, race, gender, and metastatic status), univariate and multivariate logistic regression were applied. Based on the results, only the risk score was significantly associated with overall survival in both univariate and multivariate analysis (Table 3).

**TABLE 2:** Clinicopathological variables of training and validation dataset. IQR means interquartile range.

		Training samples	Validation samples	*p*	Test	
n		117	50			
OS.time (median [IQR])		4.28 [2.28, 9.34]	4.50 [2.26, 9.44]	0.917	Kruskal–Wallis test	
OS (median [IQR])		0.00 [0.00, 1.00]	0.00 [0.00, 1.00]	0.428	Kruskal–Wallis test	
Age (median [IQR])		53.00 [44.00, 68.00]	57.50 [46.25, 69.50]	0.318	Kruskal–Wallis test	
Gender (%)	Female	51 (43.6)	18 (36.0)	0.459	Chi-square test	
	Male	66 (56.4)	32 (64.0)			
Race (%)	Asian	2 (1.7)	2 (4.0)	0.668	Chi-square test	
	Not reported	2 (1.7)	1 (2.0)			
	White	113 (96.6)	47 (94.0)			
MetStatus (%)	Metastatic	109 (93.2)	46 (92.0)	1	Chi-square test	
	Non-metastatic	8 (6.8)	4 (8.0)			
Stage (%)	I/II nos	4 (3.4)	1 (2.0)	0.197	Chi-square test	
	Not reported	16 (13.7)	2 (4.0)			
	Stage I	24 (20.5)	16 (32.0)			
	Stage II	18 (15.4)	12 (24.0)			
	Stage III	50 (42.7)	17 (34.0)			
	Stage IV	5 (4.3)	2 (4.0)			
mRNAsi (median [IQR])		0.66 [0.53, 0.73]	0.67 [0.58, 0.74]	0.362	Kruskal–Wallis test	
mDNAsi (median [IQR])		0.24 [0.17, 0.32]	0.20 [0.14, 0.29]	0.089	Kruskal–Wallis test	
Stromal score (median [IQR])		0.52 [0.37, 0.63]	0.46 [0.38, 0.57]	0.434	Kruskal–Wallis test	
Immune score (median [IQR])		0.61 [0.52, 0.74]	0.60 [0.49, 0.79]	0.969	Kruskal–Wallis test	
Tumor purity (median [IQR])		0.57 [0.42, 0.67]	0.60 [0.35, 0.71]	0.737	Kruskal–Wallis test	

**TABLE 3:** Univariate and multivariate Cox regression analyses of 11 mRNAsi-related genes signature and clinical variables associated with overall survival in subtype 2 datasets.

	Univariate analysis				Multivariate analysis			
Marker	unicox_p	HR	lower .95	upper .95	mutlicox_p	exp(coef)	lower .95	upper .95
Age	0.003	1.026	1.009	1.044	0.051	1.019	1.000	1.039
Gender	0.487	1.197	0.722	1.985	0.716	1.111	0.629	1.962
Race	0.887	1.177	0.123	11.283	0.976	0.968	0.119	7.875
MetStatus	0.065	3.202	0.930	11.021	0.065	3.512	0.926	13.314
Stage	0.186	1.154	0.933	1.428	0.985	0.998	0.784	1.270
Risk score	0.000	1.225	1.154	1.300	0.000	1.270	1.176	1.370

### Association Between Stemness Indices and Immune Microenvironment

To evaluate the associations between stemness indices and immune microenvironment, correlations analysis between immune cell individuals and mRNAsi (Figure 5D) and mDNAsi (Figure 5E) was performed. In mRNAsi, most of the immune cells were negatively correlated with mRNAsi, in which iDC, macrophages, mast cells, NK cells, TFH, and Tgd were commonly negatively correlated with three subtypes, while only Th2 cell was commonly positively correlated with three subtypes. As for mDNAsi, less immune cells were associated with mDNAsi compared to mRNAsi and only CD8 T cell and cytotoxic cell were commonly negatively associated with three subtypes.

### Immuno/Chemotherapeutic Response Prediction

**FIGURE 6:**
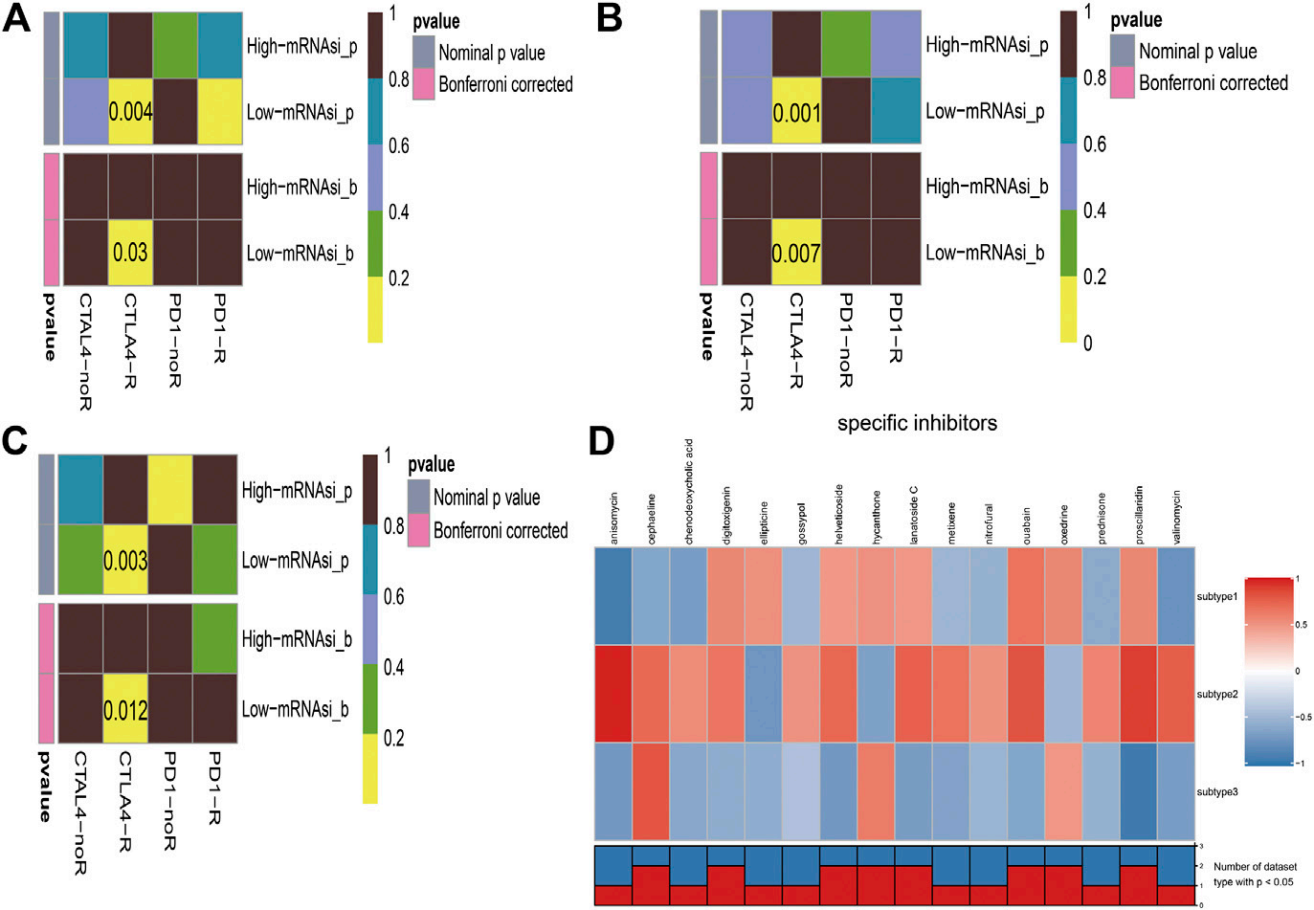
Immunotherapeutic response and potential compounds identification. **(A)**: differential immunotherapeutic response targeting CTLA-4 and PD-1 between the high- and low-risk patients in subtype 1; **(B)**: differential immunotherapeutic response targeting CTLA-4 and PD-1 between the high- and low-risk patients in subtype 2; **(C)**: differential immunotherapeutic response targeting CTLA-4 and PD-1 between the high- and low-risk patients in subtype 3; **(D)**: heatmap of potential compounds and enrichment score (positive in red, negative in blue) obtained from the Connectivity Map database for each melanoma subtype. The bottom panel showed that the number of subtypes significantly enriched in compounds.

Immunotherapy is regarded as an emerging therapy and widely used in melanoma. Therefore, we conducted the TIDE algorithm and subclass mapping to compare the expression profile of the two subgroups and another published dataset containing 47 patients with melanoma that responded to immune checkpoint inhibitors (CTLA-4 and PD-1). Interestingly, we found that the low-risk group in subtype 2 is more promising to respond to anti-CTLA-4 therapy (Bonferroni corrected *p* = 0.007) (Figure 6B). Then, we applied the same method to predict immune checkpoint inhibitors for other melanoma subtypes. We surprisingly found that low-risk groups no matter in subtype 1 (Figure 6A) or subtype 3 (Figure 6C) significantly responded to anti-CTLA-4 therapy (Bonferroni corrected *p* = 0.03; Bonferroni corrected *p* = 0.012). Moreover, chemotherapy is a common treatment for melanoma. Therefore, the Connectivity Map database was also applied to predict potential compounds. Compounds significantly correlated with at least two cancer subtypes will be selected (Figure 6D). Eventually, 16 compounds were significantly enriched, including anisomycin, cephaeline, chenodeoxycholic acid, digitoxigenin, ellipticine, gossypol, helveticoside, hycanthone, lanatoside C, metixene, nitrofural, ouabain, oxedrine, prednisone, proscillaridin, and valinomycin.

## Discussion

Worldwide, cutaneous melanoma is known as a common type of malignancy with high morbidity and mortality, while the traditional classification lacks clinical benefits and strategies for treatment are still ineffective. Therefore, in this study, we tried to establish a more evaluable classification system to help figure out better treatment choices for advanced melanoma patients. Therapies without inclusive consideration of gene transcription characters would bring treatment indeterminacy (Hamid et al., 2018). Given that, we sought to take gene expression, miRNA expression, and DNA methylation into account to partition melanoma profile and compared three clustering models. We successfully categorized melanoma patients into 3 validated subtypes. Interestingly, significant difference in overall survival time was observed among these 3 subtypes, which suggests that there exist biological relevance and distinction among subgroups. In addition, it’s generally accepted that melanoma tumors are composed of a mixture of different cell types such as cancer cells, cancer stem cells, and immune cells. We also defined the stemness indices (mRNAsi and mDNAsi) and tumor microenvironment indices (immune score, stromal score, and tumor purity) for different melanoma subtypes. The results manifested that subtype 2 with higher immune score and stromal score and lower mRNAsi and tumor purity score has the best survival time compared to other subtypes, which was consistent with our next findings that low risk of mRNAsi has longer survival than high risk. Correlation analysis also proved that intimate associations exist among these indices. Thus, our research provides a framework for exploring how the context of diverse cell types among subtypes may elucidate the observed diverse clinical outcomes and treatment effects.

Cancer cells are recently hypothesized to be derived from cancer stem cells which are closely correlated with relapse of malignant tumors, drug resistance, and metastasis. Recent studies have found that some stemness-related genes can not only initiate malignant neoplastic cascade and maintain the oncogenicity of stem cells, but also enhance the chemotherapy resistance of tumor stem cells (Chen et al., 2016; Chiou et al., 2017; Kharas and Lengner, 2017; Redmer et al., 2017). Therefore, therapeutic targeting genes associated with melanoma stem cells are urgently important. In this study, we developed and validated a robust stemness-related signature which contains 11 genes (LOC284837, OCLN, ABCC9, MEGF6, TSPYL5, RAB27B, TF, TNXB, KIAA0495, TCEA3, and KCNH5). Among these stemness-related genes, some have been identified to be associated with stem cells. For instance, TF (tissue factor) is a multifunctional membrane protein which correlates with various advanced cancers. The overexpression of TF can increase the activity of breast cancer stem cells in vitro (Shaker et al., 2017). The activated RAB27B expression will promote the secretion of colorectal cancer stem cell exosomes (Cheng et al., 2019). TSPYL5 is highly expressed in human pluripotent stem cells, and the overexpression of TSPYL5 is proven to promote cell proliferation and migration (Na et al., 2019). Moreover, KCNH5 and TCEA3 are shown to have high concentrations in mesenchymal stem cells and mouse embryonic stem cells (Cha et al., 2013; Jeong et al., 2013). Besides, the univariate and multivariate regression analysis indicated that the risk score of stemness-related signature could be regarded as an independent prognostic model in melanoma. Hence, it seems reasonable to believe that our identified stemness-related signature can be regarded as a prognostic biomarker for further clinical research. Consistent with taking advantage of integrated stemness indices to classified melanoma in our study, mounting evidence suggests that the control of melanoma stem cell could be typically administrated to melanoma patients (Luo et al., 2012; Rappa et al., 2008; Santini et al., 2012).

In this study, we explored the different immune environment of melanoma with different stemness indices. In mRNAsi of this study, we found that T helper 2 cell (Th2 cell) was the only commonly positively correlated with three subtypes of melanoma. Th2 cells are induced by interleukin 4, which can be secreted by basophils, eosinophils, mast cells, natural killer T cells, or differentiated Th2 cells (Lee et al., 2002). The main effect of Th2 cells is to activate B cell, and then humoral immunity would be stimulated by plasma cells. Nevertheless, tumor immunotherapy requires cellular immunity which is mainly activated by Th1. Both Th1 cells and Th2 cells can secrete cytokines to promote their proliferation and inhibit each other's proliferation (Saito et al., 1999). Under normal immune environment, Th1 cells and Th2 cells are in a relatively balanced state. Th2 bias signifies the imbalance of Th1/Th2. Th2 could strongly inhibit Th1 responses (Guenova et al., 2013). Th2 cells promote tumor growth and prevent tumor rejection. The bias of Th2 is regarded as one of the mechanisms of tumor immune escape. Previous research proved that the tumor microenvironment of advanced melanoma is composed of Th2-type polarization that facilitates disease progression. Studies have also shown that Th2 dominance could mediate chronic inflammation which could promote melanoma metastasis (Nevala et al., 2009). It has been reported that, in melanoma, plasmacytoid dendritic cells can break this kind of immune homeostasis by OX40L and ICOSL to support melanoma progression (Aspord et al., 2013). Reversing the imbalance of Th1/Th2 has been a concerned treatment for tumors and other diseases (Kidd, 2003). Our results further supported the importance of treatment to Th2 bias in melanoma. To date, immunotherapy is pivotal for the treatment of patients with advanced melanoma patients. Cytotoxic lymphocyte-associated antigen 4 (CTLA-4) can compete with CD28 receptor-binding antigen-presenting cell surface binding sites. CD28 receptors can activate T cells. CTLA-4 is a highly homologous molecule with CD28 and binds to the B7 molecule (CD80/CD86), and the binding strength is higher than CD28. So once CTLA-4 is highly expressed and combined, it will be a loss of the co-stimulatory signal, and then CTLA-4 would inhibit lymphocyte activation and proliferation. CTLA-4 plays a key role in regulating the T-cell system and is often used as suppressive immune molecules in tumor therapy. Anti-CTLA-4 monoclonal antibodies can augment T-cell activation and proliferation and amplify immunity by blockading CTLA-4 pathways, which enhances the patient’s ability to perform an antitumor immune response. The use of CTLA-4 monoclonal antibodies to block the CTLA-4 pathway in clinical immunotherapy of tumors also has been the current research hotspot (Carreno et al., 2000; Wells et al., 2001). However, in clinical data, the treatment has no survival benefits (Boasberg et al., 2010; Robert et al., 2011). Immune checkpoint inhibitors also have some severe side effects mostly because the blockade of the immune checkpoint pathway makes the immune responses of related organs and tissues amplified; it cannot be terminated in time, and autoimmune damage would occur. Which kind of patients is appropriate to a special treatment remains unclear. As of now, we still do not have sufficient evidence to guide clinical decisions. In this study, we comprehensively described the stemness and environmental characteristics of melanoma and found that low-risk mRNAsi groups are promising to respond to anti-CTLA-4 therapy which may provide effective measurement solutions to help the final clinical decision and hoped to help patients with advanced melanoma get the maximum remission rate.

Additionally, 16 potential compounds were identified to significantly correlate with at least two cancer subtypes. Few of these compounds have been used in melanoma researches in vitro or in vivo. For example, previous experiments proved that low doses of anisomycin can inhibit one-third of protein synthesis in melanoma cells and induce cancer cell apoptosis (Slipicevic et al., 2013). Gossypol was demonstrated to have more cytotoxic to melanoma cell lines than the conventional drugs like melphalan, cisplatin, and dacarbazine (Blackstaffe et al., 1997). What is more, nitrofural is known to act as pro-drugs, and the combination of olaparib and nitrofural will enhance the effect for the treatment of melanoma (McNeil et al., 2013). Although large part of compounds had not been reported for the treatment of melanoma, these undiscovered compounds may be regarded as the promising drug for the subsequent melanoma research.

Although our preliminary results have several implications for patients with melanoma, several limitations must be considered. Firstly, melanoma patients are recruited from public database, and findings in this research are carried out by bioinformatics methods. Secondly, the sample size in this study is small, and experimental verifications are lacking. Thus, additional fundamental researches are needed to explore the underlying mechanisms.

In conclusion, our studies provide a comprehensive cellular characterization for melanoma classification and additional subtypes that may benefit from stemness-related genes targeted therapies. Our studies also afford strategies to assess more promising population for immunotherapy and identify several potential compounds that could supply more effective treatment.

## Data Availability

The datasets presented in this study can be found in online repositories. The names of the repository/repositories and accession number(s) can be found below: TCGA-SKCM cohorts were downloaded from TCGA database (https://www.cancer.gov/tcga/). GSE65904 cohorts were downloaded from GEO database (https://www.ncbi.nlm.nih.gov/geo/).
